# 994 Alternative Route When Airplanes Don’t Fly

**DOI:** 10.1093/jbcr/iraf019.525

**Published:** 2025-04-01

**Authors:** Nichole Schiffer

**Affiliations:** Children’s Hospital Colorado Burn Center

## Abstract

**Introduction:**

Standard burn rehabilitation of the skin grafted axilla includes shoulder positioning in abduction, commonly achieved through airplane orthoses. In the pediatric population, fabricating an airplane orthosis can be challenging particularly when the patient has comorbidities. When pediatric burn patients have underlying diagnoses such as anxiety, sensory processing disorder or autism spectrum disorder, the challenge to fabricate an airplane orthosis is amplified. This method introduces a new technique to fabricate a shoulder positioning orthosis when traditional airplane fabrication proves too difficult or distressing for the patient.

**Methods:**

OT collaborated with a compression garment designer to fabricate a neoprene shirt with a sleeve and pocket. Measurements were taken of the patient similar to that of compression garment shirt. OT provided guidance to the designer on strapping, pocket sizing and placement. The pocket was designed to add thermoplastic material to appropriately position the patient. Thermoplastic was cut to fit the patient and inserted into the pocket. The neoprene shirt and thermoplastic were heated simultaneously in the splint pan. All water was pressed out of the neoprene shirt for patient safety and tolerance to molding. OT donned the neoprene shirt on the patient. The neoprene assisted with keeping the temperature of the thermoplastic away from the patient and gently molded the thermoplastic around the torso and arm while giving the patient sensory input during the process. This novel orthosis allowed for minimal handling by the therapist. The therapist held the patient with only one hand at the affected arm to support the desired axilla position.

**Results:**

This orthosis was molded successfully with minimal handling and improved tolerance and sensory experience for the neurodivergent pediatric burn patient. The orthosis was comfortable as the thermoplastic was lined with neoprene during wear. There were no skin integrity issues with wear. The patient was engaged in orthosis wear schedule and had difficulty transitioning out of it when it was discharged. Considering the patient client factors, the patient has a good outcome status-post split-thickness skin grafting to torso, axilla and upper extremity.

**Conclusions:**

When traditional rehabilitation methods do not fit our patients’ needs, we need to create alternative methods. This approach provided an alternative method of orthosis fabrication that met this patient’s sensory needs while also providing the appropriate position of the axilla for optimal healing. This novel airplane orthosis may be beneficial in pediatric patients to maintain shoulder range of motion following deep burn injury and/or skin grafting of the axilla. Improvements and adaptations may be indicated to improve efficacy of the novel shoulder orthosis.

**Applicability of Research to Practice:**

This is not a research project but is applicable to burn rehabilitation and may be best as a poster.

**Funding for the Study:**

N/A

